# Effect of harvest seasonal and gamma irradiation on the physicochemical changes in pineapple fruit cv. Pattavia during stimulated sea shipment

**DOI:** 10.1002/fsn3.485

**Published:** 2017-06-30

**Authors:** Apichai Jenjob, Apiradee Uthairatanakij, Pongphen Jitareerat, Chalermchai Wongs‐Aree, Sukanya Aiamla‐Or

**Affiliations:** ^1^ Division of Postharvest Technology School of Bioresources and Technology King Mongkut's University of Technology Thonburi Bangkok Thailand; ^2^ Learning park King Mongkut's University of Technology Thonburi Ratchaburi Thailand

**Keywords:** γ‐irradiation, low‐temperature storage, postharvest disorder, quarantine treatment

## Abstract

Gamma irradiation is used as a phytosanitary treatment for tropical fresh fruit from some producing countries. An experiment was carried out to study the effect of gamma irradiation and season of harvest on the quality ‘Pattavia’ pineapple fruit. Fruit harvested in the summer and the rainy cool (winter) seasons were exposed to gamma radiation at dose levels of 0 and 400–600 Gy from a 60 Cobalt source and the fruit stored at 13°C and 90% RH for up to 21 days. Gamma irradiation did not affect the ratio of TSS/TA, antioxidant content, or ascorbic acid concentration. However, gamma irradiation did delay color development and also induced internal browning over 50% of flesh discolored in fruit stored for 14 days, especially harvested winter fruit. Moreover, harvesting fruit in different seasons had a significant effect on fruit quality after harvest and during stimulated sea shipment storage. The result showed that gamma irradiation can be used as a phytosanitary treatment with minor changes in eating quality. However, the internal browning was greater if fruit were stored longer than 1 week at 13°C.

## 1 INTRODUCTION

Insect quarantine barriers require commercial phytosanitary treatments that include thermal treatments (hot water dip or vapor treatment) or irradiation. Gamma irradiation has emerged as a potential economic alternative for insect disinfestations, sterilization, inhibition of sprouting, and for the extension of the storage life of fresh fruits and vegetables (Lacroix, 2007). Thailand is an important producer of pineapple, especially canned fruit, but is also attempted to increase the export fresh fruit to the US market. Entry of Thai pineapple into the US market requires a phytosanitary gamma irradiation treatment to sterilize potential insect pests. Several studies have investigated the application of irradiation treatments to disinfest various fruits (Camargo, Tadini, & Sabato, 2007; Cetinkaya, Ozyardimic, & Denli, 2006; Follett, 2004; Hallman, 1999, 2000; Paul & Rohrbach, 1985). Radiation treatment reduced ascorbic acid and β‐carotene in guava with not significant changes in sugars, pectin, and citric acid (Rosario, Julieta, Emilia, & Valdivia‐Lopez, 2013). The total phenolic content and antioxidant capacity of raspberries increases with radiation doses (Verde et al., 2013). Titratable acidity, total soluble solids, and chroma are significantly (*p* ≤ .05) less in early harvest pears and the hue is increased by irradiation suggesting that there are differences in radiotolerance of early and late harvest pears (Abolhassani, Caporaso, Rakovski, & Prakash, 2013). However, ascorbic acid and titratable acidity in mango and papaya are only slightly changed during ripening with no difference in the total sugar content between the irradiated and non‐irradiated fruit (Thomas & Beyers, 1979). Gamma irradiation at doses up to 0.6 kGy had no effect on skin or flesh color and soluble solids content of both Nam Dokmai and Chok Anan mango cultivars (Uthairatanakij, Jitareerat, & Kanlayanarat, 2006).

Internal browning (IB) or blackheart is the most important physiological disorder of pineapple that is induced by exposure to low temperature, either preharvest or postharvest (Hallman, 2000; Akamine, Goo, Steepy, Greidanus, & Iwaoka, 1975; Youryon, Wongs‐Aree, McGlasson, Glahan, & Kanlayanarat, 2011; Smith, 1983). ‘Pattavia’ pineapple rapidly develops IB within 2–3 days after the fruit are transferred to ambient temperatures (20°C). Peng, Cao, & Zheng (2013) demonstrated that chilled‐pineapple fruit show increased ethylene production. Moreover, our earlier studies revealed that gamma irradiation also induced the internal browning in ‘Trad Si Thong’ pineapple fruit (Uthairatanakij, Jitareerat, Srilaong, & Photchanachai, 2013). In addition, inconsistency occurs in the incidence and severity of IB symptoms between different experiments (Uthairatanakij et al., unpubl. data). The objective of this study was to evaluate the effect of the gamma irradiation on ‘Pattavia’ pineapple fruit harvested in winter and summer seasons during sea shipment stimulation.

## 2 MATERIALS AND METHODS

### 2.1 Plant materials and gamma irradiation treatment

Pineapples (*Ananas comosus* L. cv. Pattavia) harvested from East Thailand in the summer (April, 2014) and rainy cool (winter) seasons (December, 2013) were transported to our laboratory within three hours. Fruit of uniform shape, size, color, and weight were selected, washed with tap water, dipped into Carbendazim fungicide solution (1,000 ppm, 3 min) to control postharvest decay, and then air dried at 25 °C for 2 h. All fruit were packed in carton (ca. 6–7 kg) and kept at 13°C for 1 day before irradiation. Fruit were divided into two groups; the first group was subjected to gamma irradiation in a cobalt‐60 irradiator at Thailand Institute of Nuclear Technology, Nakornnayok Province, Thailand with 400–600 Gy absorbed doses in the fruit, based upon the carton location in the irradiator. The source strength was approximately 430 kCi. Dosimetry was performed using 3‐mm‐diameter FWT‐70 series (Opti‐Chromic Dosimeter). During treatment, dosimeters were placed at minimum and maximum dose locations previously determined by dose mapping. Midway through the treatment, the boxes were rotated 180° to ensure a more uniform treatment. Another group was subjected to the same conditions without irradiation. Following treatment, all fruit (treated and control fruit) were kept under refrigerated storage conditions (13°C, 80–90% RH) to simulate shipment by sea to the United States. Fruit were evaluated for physicochemical changes in internal browning (IB), quality parameters (total soluble solids, titratable acidity, and color), antioxidant level, and molondialdehyde (MDA) content immediately upon arrival of the fruit from the field. Ten fruit were randomly sampled and quality evaluated after 1, 7, 14, and 21 days of storage at 13°C. For ascorbic acid, antioxidant activity, and MDA content, the fruit tissues from the equatorial area of each sample were frozen in liquid nitrogen and stored at −20°C until use. All assessments were conducted with three replicates.

### 2.2 Fruit quality analysis

The total soluble solids (TSS) of fruit juice were determined using a digital hand‐held refractometer (0–52°Brix; Atago PAL‐1, Japan). The results are reported as percent total soluble solids at 25°C. Titratable acidity (TA) of fruit juice was assayed based on the method of Uthairatanakij, Holford, & Mcglasson (2005) Briefly, 5 g of pineapple flesh was extracted and titrated with 0.1‐N sodium hydroxide using phenolphthalein as the indicator and expressed as a percentage of citric acid. Color of fruit peel and pulp was measured using a colorimeter (CR 300, Minolta, Japan). The measurements are expressed as hue angle (actual color). Three readings were taken at three locations on each fruit and the average values calculated.

Internal browning (IB) and translucent intensities were evaluated according to a slight modification in Teisson's method (Teisson, Combres, Martin, & Marchal, 1979). The fruit were cut longitudinally in halves and for each fruit, IB and translucent intensities were scored from 0 to 5 according to the percentage of flesh affected from 0—free from those symptoms; 0.5—watery spots at the base of the fruitlets; and 1–5—<10, 11–25, 26–50, 51–75, and >75% of the flesh discolored, respectively.

### 2.3 Determination of malondialdehyde content (MDA)

MDA content was determined according to the method of Heath and Packer (Heath & Packer, 1968). Frozen flesh tissue (5 g) was homogenized with 25 mL of ice‐cold 5% trichloroacetic acid (TCA). The homogenate was centrifuged at 28,000 *g* for 15 min at 4°C. A 2‐ml aliquot of the supernatant was thoroughly mixed with 2 ml of 0.67% 2‐thiobarbituric acid (TBA) in 20% TCA. The mixture was heated at 100°C for 15 min and then quickly cooled in an ice bath for 5 min. After that, the mixture was centrifuged at 28,620 *g* for 15 min at 4°C. The absorbance of the supernatant was measured at 532 nm using a UV–visible spectrophotometer (Shimadzu, Japan). The value of the nonspecific absorption at 600 nm was subtracted. The MDA content was calculated using the extinction coefficient of 155 mmol L^−1^ cm^−1^ and was expressed as μmol per kilogram of fresh weight (FW).

### 2.4 Determination of ascorbic acid

The determination of ascorbic acid (AA) was conducted according to AOAC adapted by Oliveira, Godoy, & Prado (2010) (AOAC, 1997). The extracts (500 mg) were homogenized in 50 mL of aqueous solution of 1% metaphosphoric acid and submitted to titration using 2, 6‐dichlorophenol‐indophenol. Ascorbic acid of fruit flesh was measured based on the method described by Li, Zhao, & Tang (2010). The results were expressed as mg per kilogram of FW. A standard AA solution was prepared in range 10–80 mg/mL.

### 2.5 Determination of total phenolic content

The total phenolic content of the pulp was measured using a Folin–Ciocalteu colorimetric method (Singleton, Orthofer, Lamuela‐Ravento, & Lester, 1999). The calibration curve of gallic acid was prepared 10–200 mg/ml. Absorbance was measured at 750 nm versus a blank after 60 min at room temperature. The results were expressed as milligrams of gallic acid equivalents per kilogram of fresh fruit weight.

### 2.6 Statistical analysis

All data were analyzed by analysis of variance (ANOVA) using SAS (SAS Institute, Inc., Cary, NC), and significant difference (*p* < .05) among means was determined using Duncan's multiple range test.

## 3 RESULTS

### 3.1 Effect of harvesting time on postharvest quality of pineapple fruits

At harvest time, the TSS/TA ratio of fruit harvested in summer and winter was 4.7 and 1.24, respectively. Hue angle of peel of fruit harvested in summer and winter seasons was 104.84 and 98.30, whereas hue angle of pulp was 97.52 and 97.07, respectively. The MDA content of flesh in summer and winter was 0.86 and 0.72 μmol/kg FW, respectively. In addition, the ascorbic acid contents in summer and winter harvested fruit were 7.0 and 6.8 mg/kg FW, respectively (Table 1).

**Table 1:** Effect of harvest season on ‘Pattavia’ pineapple fruit quality

Harvesting season	H value		TSS/TA ratio	MDA (μmol/kgFW) in pulp	Ascorbic acid (mg/kgFW) in pulp
	Peel	Pulp			
Summer	104.84^a^	97.52	1.24^b^	0.86	0.70
Winter	98.30^b^	97.07	4.77^a^	0.72	0.68

Mean values in the same column with different superscript letters are significantly different (*p* < 0.05).

### 3.2 Effect of harvest season on postharvest quality

**Figure 1:**
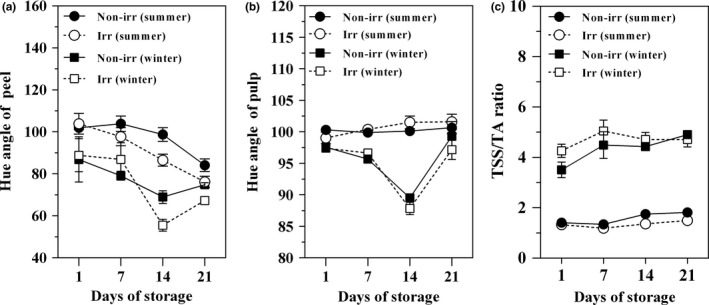
Effect of gamma irradiation on peel color (a), pulp color development (b), and TSS/TA ratio (c) of pineapple cv. Pattavia harvested during the summer and winter seasons. Fruit were irradiated with gamma radiation at 0 (Non‐irr) or 400 Gy (Irr) and then stored at 13°C

The hue angle of the pineapple fruits peel was significantly affected by harvest season and showed a higher value in summer (76.17–103.90) than winter (55.54–88.87) harvested fruit during storage (*p* < .05) (Figure 1a). Gamma irradiation significantly affected the hue angle of peel after 14 days of storage (*p* < .05). On 14th day of storage, summer harvested fruit was highest hue angle of peel (98.75), whereas harvested winter fruits with gamma irradiation were lowest hue angle of peel (55.54). After storage at 13°C, the hue angle tended to decrease in all treatments throughout the storage period. Nongamma irradiated fruit harvested in summer (84.11) showed the highest hue angle when compared to winter harvested fruit (74.80).

The hue angle of the pulp was significantly affected by harvesting time and gamma irradiation (*p* < .05). During storage, summer harvested fruit irradiated with gamma radiation had the highest hue angle (99.03–101.59), whereas fruit harvested in winter and irradiated with gamma ray had the lowest hue angle (87.84–97.40) (Figure 1b).

TSS/TA ration was trendily increased during storage. Harvest season had a significant effect on the TSS/TA ratio of pineapple with winter harvested fruit (4.50) having a higher TSS/TA ratio than summer harvested fruit (1.45) (*p* < .05). However, gamma irradiation did not affect TSS/TA ratio when compared to untreated fruit (Figure 1c). Fruit harvested in winter and irradiated with gamma radiation had the highest TSS/TA ratio, whereas summer irradiated fruit had the lowest TSS/TA ratio (*p* < .05).

### 3.3 Effect of harvest season on IB and translucency

**Figure 2:**
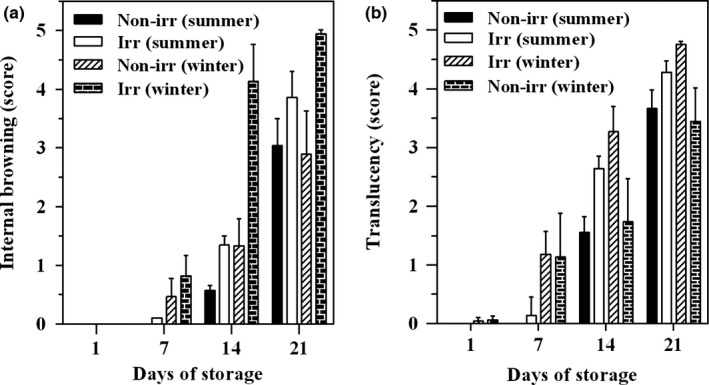
Effect of gamma irradiation on the internal browning (a) and translucency (b) of pineapple cv. Pattavia harvested during the summer and winter seasons. Fruit were irradiated with gamma radiation at 0 (Non‐irr) or 400 Gy (Irr) and then stored at 13°C

The internal browning of pineapple in all treatments occurred near the core at the base of the fruitlets and it was observed only slightly on day 7 (0.10–0.80) then rapidly increased up to day 21 (2.89–4.94) of storage with significant difference among treatments (*p* < .05) (Figure 2a). IB symptoms in winter harvested irradiated fruit were higher than the summer harvested fruit. Gamma irradiated fruit showed higher IB severity than nontreated fruit. IB symptoms in the nontreated pineapple appeared over 50% of the surface after 21 days of storage, whereas the gamma irradiated fruit harvested in winter occurred over 50% in 14 days (Figure 2a).

Pineapple fruit harvested in winter showed higher signs of flesh translucency than summer harvested fruit. In addition, flesh translucency was higher for fruit irradiated with gamma ray than nontreated fruit (Figure 2b). Flesh translucency was exhibited on day 7, except for summer harvesting fruit, with the difference in severity becoming more noticeable after 14 days of storage. At day 14, the value of flesh translucency of gamma irradiation samples reached a score of 3.2, whereas no sign of flesh translucency could be detected by visual inspection of nonirradiated fruit harvested in summer. This symptom progressively increased as storage duration increased.

### 3.4 Effect of harvesting time on the biochemical change in gamma irradiated pineapple fruits

**Figure 3:**
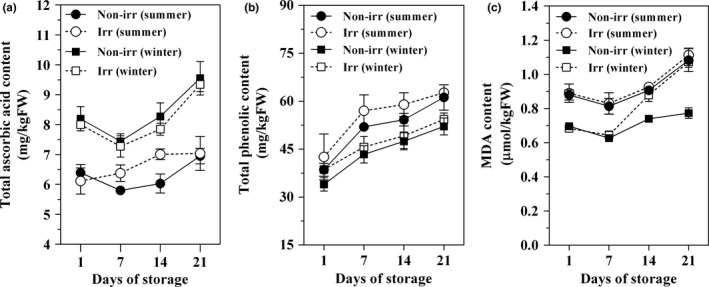
Effect of gamma irradiation on total ascorbic acid (a), total phenolic (b), and malondialdehyde (MDA) contents (c) of pineapple cv. Pattavia harvested during the summer and winter seasons. Fruit were irradiated with gamma radiation at 0 (Non‐irr) or 400 Gy (Irr) and then stored at 13°C

Total ascorbic acid content was increased from 6.20 to 7.03 mg/kgFW and 8.10 to 9.55 mg/kgFW in summer and winter harvested fruits, respectively. Moreover, total ascorbic acid content in the flesh of pineapple harvested in winter was significantly higher than that of summer harvesting fruit (*p* < .05). Conversely, gamma irradiation did not affect the total ascorbic acid in flesh (Figure 3a). During storage, the total phenolic content in the flesh was higher in the winter harvested (41.10–61.50 mg/kgFW) than summer harvested (33.50–53.1 mg/kgFW) fruit (Figure 3b). However, the total phenolic content was not affected by gamma irradiation.

Changes in the MDA content of gamma irradiated and non‐irradiated flesh were shown in Figure 3c. The amount of MDA content in summer harvesting fruit was markedly higher than that of winter harvested fruit. The content of MDA tended to increase during storage. At 21 days, the MDA content in flesh was in the range 0.67–1.13 μmol/kg FW.

## 4 DISCUSSION

Physical appearance such as peel and pulp color is important sensory attributes related with the ripening quality of fruit. Significant difference occurred in peel color of fruit harvested in summer with a higher hue angle value than that of winter harvested fruit. Gamma irradiation inhibited peel degreening. Fruit harvested in summer and not treated had the highest hue angle, with the hue value declining during storage. Moreno et al. (2006) reported that radiation induced a greener peel and lighter pulp color of mango fruit. The color change during fruit ripening is due to the degradation of chlorophyll (Hortensteiner & Krautler, 2011).

Taste development in fruit is due to a general increase in sweetness and decrease in organic acids, resulting in an optimum consumer preferred sugar/acid ratio (Lizada, 1993). This study showed that the ratio of TSS/TA in the pineapple fruit subjected to gamma irradiation treatment tended to increase, but was not significant when compared to the untreated fruit throughout the entire storage period. However, the fruit harvested in winter had a higher TSS/TA ratio than summer harvested fruit (Figure 1c). The data indicated a TSS/TA ratio increase due to the amount of TA decrease and TSS increase as a result of seasonal harvesting. Perecin, Silva, Harder, Oliveira, & Arthur (2011)found that the variation in the soluble solids content of irradiated minimally processed pineapples was a function of the radiation dose and storage period. A slower rate of sugar accumulation was found in irradiated mango fruit (Singh, 1990). The interaction of location and season seems to be more profound on cactus pear fruit sugar and acidity levels (Shongwe, De Wit, Osthoff, Nel, & Labuschagne, 2013).

IB (Figure 2a) and translucency (Figure 2b) were the major physiological disorders observed after pineapple fruit were stored at 13°C. The results showed that gamma irradiation induced either or both flesh browning or translucency. Tissue browning has been related to physiological stress induced by high irradiation doses (Frylinck, Dubery, & Schabort, 1987). Browning is associated with an increased activity polyphenol oxidase and phenylalanine ammonia lyase (Saltveit, 2000). Gamma irradiation does increase polyphenol oxidase activity via enzyme activation rather than a de novo synthesis (Thomas & Nair, 1971). Flesh browning induced by irradiation has also been reported in ‘Gala’ apple fruit and ‘Zebda’ mango (Fan & Mattheis, 2001; Youssef, Asker, El‐Samahy, & Swailam, 2002). In addition, translucency is correlated with the cell wall invertase activity due to sucrose accumulation during fruit development and its severity increased with fruit development (Chen & Paull, 2000).

Fruit and vegetables are the predominant sources of vitamin A, C, E, and thiamin (Kilcast, 1994). There was a slight decrease in the total ascorbic acid content of all treatments. Total ascorbic acid in the flesh of pineapple harvested in winter was significantly higher than that of summer harvesting fruit. However, fruit irradiated at 0 and 0.4 kGy did not differ in ascorbic acid content (Figure 3a). Gamma irradiation has been shown to cause a decline in the ascorbic acid content of strawberry and ‘Autumn Bliss’ raspberries (Hussain, Dar, & Wani, 2012; Tezotto‐Uliana, Berno, Saji, & Kluge, 2013). Seasonal variation in ascorbic acid content has been reported in tomato, raspberries, and oranges (Pirogovskaia, Kempler, Kitts, & Lund, 2012; Rosello, Adalid, Cebolla‐Cornejo, & Nuez, 2011; Dhuique‐Mayer, Fanciullino, Dubois, & Ollitrault, 2009). Under stress conditions including irradiation, ROS generation often exceeds the overall cellular antioxidative potential leading to physiological and biochemical changes that adversely affect plant physiology and biochemistry (Ahmad, Sarwat, & Sharma, 2008). Figure 3 showed that the pineapple flesh antioxidant system in terms of ascorbic acid and total phenolic content was not affected by gamma irradiation. Several reports confirm that enhanced antioxidant defense combats the oxidative stress induced by abiotic stressors such as UV radiation (Li et al., 2010; Kumari, Singh, & Agrawal, 2010) and gamma irradiation (Kavitha et al., 2015). An increase in phenolic compound biosynthesis and accumulation frequently occurs in plant tissues as a reaction to biotic and abiotic stresses (Dixon & Paiva, 1995). Therefore, plants possess an efficient nonenzymatic antioxidant defense system such as ascorbic acid and phenolic compounds that limit uncontrolled oxidation and protect plant cells from oxidative damage by scavenging ROS (Ahmad et al., 2008; Kumari et al., 2010; Kavitha et al., 2015; Dixon & Paiva, 1995; Mittler, Vanderauwera, Gollery, & Van Breusegem, 2004). Malondialdehyde (MDA), an indicator of lipid peroxidation caused by ROS, tended to increase during storage and the contents were higher in fruit harvested in summer (Figure 3c). Similar results were found in mume fruit (Imahori, Takemura, & Bai, 2008). Pineapple fruit harvested in summer had a higher oxidative stress due to lower temperature and lower solar irradiance during fruit development resulting in a higher MDA content (Prado, Prado, Pagano, & Rosa, 2015).

## 5 CONCLUSIONS

In conclusion, this study indicated that the application of gamma irradiation to disinfest ‘Pattavia’ pineapple fruit induced IB, but did not affect other components of postharvest quality (color development and TSS/TA ratio) during stimulated sea shipment, but harvest season did impact quality. Therefore, this study contains useful commercial information and points to further research needs to reduce the effect of gamma irradiation on pineapple fruit quality.

## CONFLICT OF INTEREST

None declared.
